# The Ablation Properties of CO_**2**_ Laser Irradiating to Absorption Media: An In Vitro Study

**DOI:** 10.1155/2012/230967

**Published:** 2012-11-25

**Authors:** Sajee Sattayut, Kittiwut Hortong, Chorpaka Kitichaiwan

**Affiliations:** ^1^Lasers in Dentistry Research Group and Oral Surgery Department, Faculty of Dentistry, Khon Kaen Univerisity, Khon Kaen 40002, Thailand; ^2^Dental Division, Prasat Hospital, Prasat, Surin 32140, Thailand; ^3^Dental Division, Yangtalad Hospital, Yangtalad, Kalasin 46120, Thailand

## Abstract

This study aimed to compare histological affected zone of tissue samples irradiated by defocused CO_2_ laser at 1, 2, and 3W continuous wave with and without absorption media. The in vitro experiment was conducted in 70 tissue blocks. The samples were randomly allocated into 7 groups: 10 samples each group, namely, the groups irradiated with 1, 2, and 3W, defocused CO_2_ laser for 5 seconds, the groups irradiated with 1, 2, and 3W, defocused CO_2_ laser to the absorption media, and the media alone group as a control. Then the samples were stained with Masson's trichrome and measured the affected borders under light microscope at 10 × 10 magnification. There was no histological alteration in the groups irradiated with the defocused CO_2_ laser to the absorption media while the groups without using the absorption media showed the tissue alteration by photoablation.

## 1. Introduction

 The uses of CO_2_ Laser in oral soft tissue surgery for benign soft tissue lesions [1–4] and potentially malignant disorders [5–8] were widely reported. These studies showed clearly the advantages of CO_2_ laser in terms of precise and haemostatic ablation and proving less postoperative pain, swelling, and scare formation. The favorable healing of oral soft tissue after CO_2_ laser surgery was explained by the mechanism of healing with less inflammatory reaction and fewer myofibroblasts compared with scalpel excision [9]. The immunohistological study by Zeinoun et al. [10] also found that the myofibroblast response and activity were slower and lack of contractile compared with the scalpel wound. By comparison with other type of laser such as diode laser and Nd-YAG laser, the CO_2_ laser showed the narrow area of lateral-thermal damage [11, 12] leading the shorter period of healing and the less wound contraction.

 In 2004, Sharon-Buller and Sela [13] reported the technique of using CO_2_ laser irradiating transparent gel, acting as energy absorption, resulting in immediate pain relief in patients with oral ulcer. This, as the authors referred to be a nonablative photoreaction, differed from other laser-applications which were stated in the review [4]. However, the histological ablation properties of this technique have not been explored. Therefore, this study aimed to compare histologically affected borders of the tissue samples irradiated by defocused CO_2_ laser at 1, 2, and 3W with and without transparent gel covering the tissue surface.

## 2. Materials and Methods

The laboratory experiment was conducted in 70 tissue blocks of 1 × 1 × 1 cm ventral mucosa of the fresh pig tongues. The samples were randomly allocated into 7 groups, 10 samples each group as follows:

 group 1: 1W defocused CO_2_ laser continuous wave irradiating the tissue for 5 seconds,
 group 2: 1W defocused CO_2_ laser continuous wave irradiating the absorption media on tissue surface for 5 seconds,
 group 3: 2W defocused CO_2_ continuous wave laser irradiating the tissue for 5 seconds,
 group 4: 2W defocused CO_2_ continuous wave laser irradiating the absorption media on tissue surface for 5 seconds,
 group 5: 3W defocused CO_2_ continuous wave laser irradiating the tissue for 5 seconds,
 group 6: 3W defocused CO_2_ laser continuous wave irradiating the absorption media on tissue surface for 5 seconds,
 group 7: Applying absorption media on tissue surface for 5 seconds.

### 2.1. The Sample Preparation

The samples were prepared based on the standard tissue block preparation for gross and histological study into the effect of high-intensity laser as used in the other studies [14, 15]. The fresh pig tongues were frozen in 4°C immediately after sacrificed and undertaken in the experiment within 24 hours. This can avoid the cell autolysis [16].

### 2.2. The Absorption Media

Based on Sharon-Buller and Sela [13] study, the absorption media must be transparent and mainly composed of water which highly absorbs CO_2_ laser. They used Elmex gel, high fluoride concentration gel as the media. We used Sore mouth gel, 20% bezocaine, because this was a transparent gel recommended to be used intraorally.

### 2.3. The CO_2_ Laser Machine and Its Irradiation

**Figure 1:**
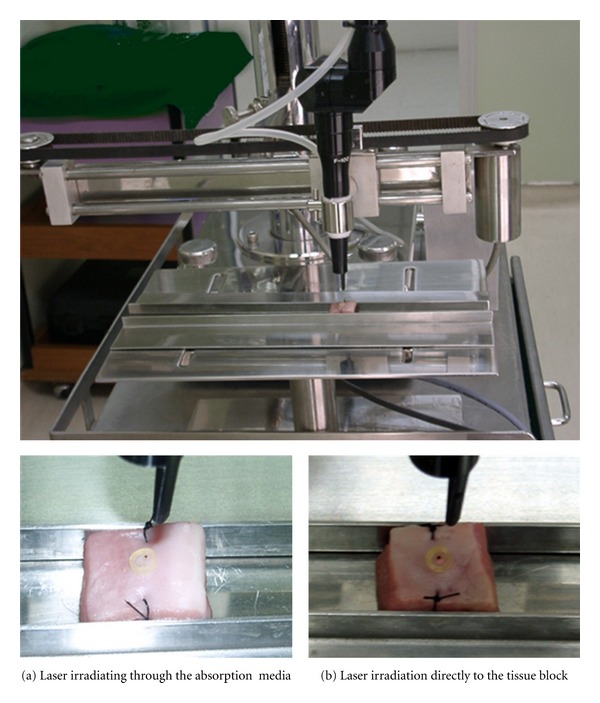
Laser irradiating to the samples.

The 10.6- micron CO_2_ laser (Smart pulse CO_2_, Model: SNJ-1000, Korea) with adjustable power from 1 to 25W and 0.3 mm focal spot-diameter with articulated arm optical delivery was used in this experiment. The regimens were 1, 2, and 3W continuous wave at 2-time defocal length and 5-second irradiation with and without absorption media (Figure 1). The actual powers of the settings were measured by using optical power meter (THORLAB inc model D3MM). These were the same amount of powers which were on the surface of the samples. The measurement of the actual powers and theirs calculated fluences were shown in Table 1. 

### 2.4. The Experimental Methods

1. The samples were sutured with 3-0 black silk at both margins for locating the central point and placed on the customized apparatus. The ventral mucosa was used for the experiment.
2. The samples were randomly allocated into 7 groups as follows: Groups 1, 3, and 5 were irradiated with defocused CO_2_ laser for 5 seconds at 1, 2, and 3W, respectively.
3. Groups 2, 4, and 5 were applied with absorption media gel on the surfaces using the template; 5 mm diameter and 1 mm thickness, and then irradiated with defocused CO_2_ laser for 5 seconds at 1, 2, and 3W, respectively.
4. Group 7 was applied with absorption media gel on the surfaces using the template, 5 mm diameter and 1 mm thickness for 5 seconds.
5. All samples were strained with Masson'trichrome and then inspected under light microscope at 10 × 10 magnification.

### 2.5. Histological Measurement

**Figure 2:**
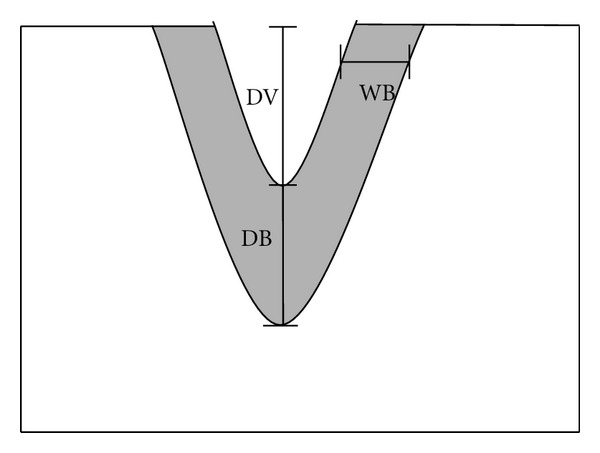
The measurements of the histologically affected borders. Depth of vaporization: DV. Depth of vertically affected border: DB. Width of horizontally affected border: WB.

 According to the Masson's trichrome stain, the affected collagen by laser was indicated in red band [14, 17]. The borders of histological changes (Figure 2), namely, depth of vaporization (DV), depth of vertically affected border (DB), and width of horizontally affected border (WB) were measured in micron. The measurements were undertaken by 2 inspectors under double-blind randomized controlled trial. The before and after calibrations were conducted. 

### 2.6. Data Analysis

 The normality test was calculated using Shapiro-Wilk test. The data was described using descriptive statistics and compared with the groups using ANOVA and Tukey test multiple comparison. In case, the data was not in normal distribution, Kruskal Wallis would be applied.

## 3. Results

**Figure 3:**
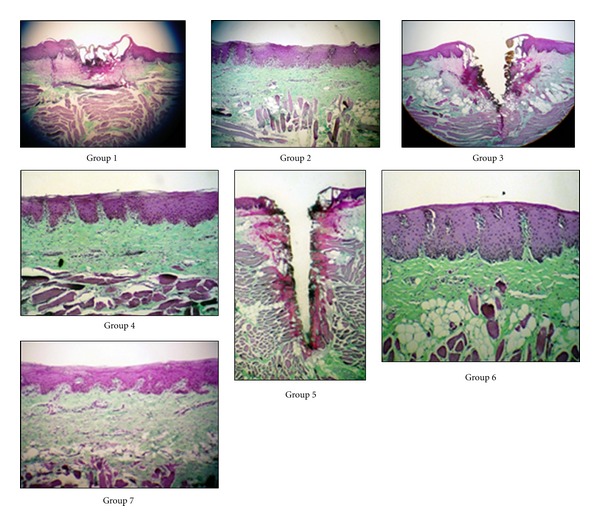
Histological finding of the groups at 10 × 10 magnification. Group 1: 1W defocused CO_2_ laser irradiation. Group 2: 1W defocused CO_2_ laser irradiation with absorption media. Group 3: 2W defocused CO_2_ laser irradiation. Group 4: 2Wt defocused CO_2_ laser irradiation with absorption media. Group 5: 3W defocused CO_2_ laser irradiation. Group 6: 3W defocused CO_2_ laser irradiation with absorption media. Group 7: Absorption media alone.

The data was in normal distribution. The intraclass correlation coefficient at 0.8 showed the *P* value being less than 0.001. Therefore, parametric statistics was used for analysis. There was no histological affected area in the groups irradiated with the defocused CO_2_ laser to the absorption media while the histological changes were found in the groups irradiated with CO_2_ laser directly (Figure 3 and Table 2). 

The comparison of the measurements was shown in Table 3. The group irradiated with 3W defocused CO_2_ laser had statistically larger depth of vaporization than the groups irradiated with the defocused 1 and 2W defocused CO_2_ (*P*  value = 0.001 and 0.013). The mean differences were 762.48 microns (95%  CI = 287.26 to 1,237.70) and 588.71 microns (95%  CI = 112.89 to 1,063.33), respectively. The depth of affected border of the 3W defocused CO_2_ group was larger than the 1 and 2W defocused CO_2_ groups (*P*  value = 0.001 and 0.016). The mean differences were 410.10 microns (95%  CI = 157.95 to 662.26) and 302.16 microns (95%   CI = 50.00 to 554.32), respectively. The width of affected border of the 1W defocused CO_2_ group was narrower than the 2 and 3W defocused CO_2_ groups (*P*  value < 0.001). The mean differences were −218. 17(95%  CI = −142.94  to −293.40) and −247.93 microns (95%  CI = − 323.15 to −172.70), respectively.

## 4. Discussion

There was no detection of histological alteration of the all samples in the groups irradiated with defocused CO_2_ laser to the absorption media. These were inspected under light microscope at 10 × 10 magnification. Owing to the fact that the actual laser power could not be detected by the optical power meter in the setting of defocused 1W CO_2_ laser irradiation, the regime of either 2W or 3W defocused CO_2_, of which laser power detected, is recommended for clinical application.

It was noticed that the 3W defocused CO_2_ laser irradiating to the media as used in this research was able to transfer the higher power than the 1W defocused CO_2_ laser irradiating directly without providing the ablative effect. It can be hypothesized that using this method the temperature of the tissue was not raised to the coagulative level of 50 to 60°C [18, 19]. Therefore, the clinical effect of this technique on pain control and wound healing reported by Sharon-Buller and Sela [13] tended to be related to low intensity laser inducing biomodulation [20]. 

 In terms of application of defocused CO_2_ laser for tissue vaporization, the group irradiated with 3W defocused CO_2_ laser had larger depth of vaporization and depth of vertically affected borders than the others, while the group irradiated with 1W defocused CO_2_ had less width of horizontal affected area than the others. 

## 5. Conclusion

 Histological changes were found in the groups irradiated with 1, 2, and 3 W defocused CO_2_ laser continuous wave for 5 seconds. The group irradiated with 3 watts CO_2_ laser continuous wave had larger depth of vaporization and depth of vertically affected border than the others, while the group irradiated with 1 watt had less width of horizontal affected area than the others. The 2 and 3 W defocused CO_2 _laser continuous waves irradiating for 5 seconds through the absorption media, transparent high-water content gel, can deliver the energy to the surface of tissue without causing ablation.

## Figures and Tables

**Table 1:** The actual powers of CO_2_ laser measured by the optical power meter.

Regimes	Mean Power (mW)	Standard deviation	95% confident interval (mW)	Calculated fluence (J/cm^2^)
1 Watt	20.5	8.87	16.35 to 24.65	146.43
2 Watt	233.5	25.40	221.61 to 245.39	1,667.86
3 Watt	642.5	42.41	622.65 to 662.35	4,589.29
1 Watt with laser absorption media	0	0	0	0
2 Watt with laser absorption media	15.5	9.99	10.83 to 20.17	110.71
3 Watt with laser absorption media	118	25.87	105.89 to 130.11	842.86

*Spot area: 0.0007 cm^2^.

**Table 2:** The histologically affected borders by the groups.

Group	Depth of vaporization (DV)			Depth of vertically affected border (DB)			Width of horizontally affected border (WB)		
	mean	SD	95% CI	mean	SD	95% CI	mean	SD	95% CI
1	218.56	195.39	78.79 to 358.34	287.81	132.95	192.70 to 382.91	369.15	60.76	325.68 to 412.61
2	0	0	0	0	0	0	0	0	0
3	392.92	207.13	244.76 to 541.10	395.75	163.88	278.52 to 512.98	587.32	79.39	530.52 to 644.11
4	0	0	0	0	0	0	0	0	0
5	981.04	685.53	490.64 to 1471.44	697.91	332.58	460 to 935.82	617.07	61.75	572.9 to 661.25
6	0	0	0	0	0	0	0	0	0
7	0	0	0	0	0	0	0	0	0

SD: standard deviation, 95% CI = 95% confident interval.

Group 1: 1 W defocused CO_2_ laser irradiation.

Group 2: 1 W defocused CO_2 _laser irradiation with absorption media.

Group 3: 2 W defocused CO_2_ laser irradiation.

Group 4: 2 Wt defocused CO_2_ laser irradiation with absorption media.

Group 5: 3 W defocused CO_2_ laser irradiation.

Group 6: 3 W defocused CO_2_ laser irradiation with absorption media.

Group 7: Absorption media alone.

**Table 3:** The comparisons of the differences of histologically affected borders by the groups.

Affected border	Group	Compared group	Mean difference	95% CI of the differences	*P* value
DV	1 Watt	2 Watt	−174.37	−649.58 to 300.85	0.639
		3 Watt	−762.48	−1237.70 to −287.26	0.001*
	2 Watt	1 Watt	174.37	−300.85 to 649.58	0.639
		3 Watt	−588.71	−1063.33 to −112.89	0.013*
	3 Watt	1 Watt	762.48	287.26 to 1237.79	0.001*
		2 Watt	588.71	112.89 to 1063.33	0.013*

DB	1 Watt	2 Watt	−107.94	−360.09 to 144.21	0.546
		3 Watt	−410.10	−662.26 to −157.95	0.001*
	2 Watt	1 Watt	107.94	−144.21 to 360.09	0.546
		3 Watt	−302.16	−554.32 to −50.01	0.016*
	3 Watt	1 Watt	410.10	157.95 to 662.26	0.001*
		2 Watt	302.16	50.01 to 554.32	0.016*

WB	1 Watt	2 Watt	−218.17	−293.40 to −142.94	<0.001*
		3 Watt	−247.93	−323.15 to −172.70	<0.001*
	2 Watt	1 Watt	218.17	142.94 to 293.40	<0.001*
		3 Watt	−29.76	−104.98 to 45.47	0.595
	3 Watt	1 Watt	247.93	172.70 to 323.15	<0.001*
		2 Watt	29.76	−45.47 to 104.98	0.595

*: *P*-value < 0.05.

Depth of vaporization: DV.

Depth of vertically affected border: DB.

Width of horizontally affected border: WB.
